# Detrended Fluctuation Analysis in the prediction of type 2 diabetes mellitus in patients at risk: Model optimization and comparison with other metrics

**DOI:** 10.1371/journal.pone.0225817

**Published:** 2019-12-18

**Authors:** Ana Colás, Luis Vigil, Borja Vargas, David Cuesta–Frau, Manuel Varela

**Affiliations:** 1 Department of Internal Medicine, Hospital 12 de Octubre, Madrid, Spain; 2 Department of Internal Medicine, Hospital Universitario de Móstoles, Móstoles, Madrid, Spain; 3 Technological Institute of Informatics, Universitat Politècnica de València, Alcoi Campus, Alcoi, Spain; Northumbria University, UNITED KINGDOM

## Abstract

Complexity analysis of glucose time series with Detrended Fluctuation Analysis (DFA) has been proved to be useful for the prediction of type 2 diabetes mellitus (T2DM) development. We propose a modified DFA algorithm, review some of its characteristics and compare it with other metrics derived from continuous glucose monitorization in this setting. Several issues of the DFA algorithm were evaluated: (1) Time windowing: the best predictive value was obtained including all time-windows from 15 minutes to 24 hours. (2) Influence of circadian rhythms: for 48-hour glucometries, DFA alpha scaling exponent was calculated on 24-hour sliding segments (1-hour gap, 23-hour overlap), with a median coefficient of variation of 3.2%, which suggests that analysing time series of at least 24-hour length avoids the influence of circadian rhythms. (3) Influence of pretreatment of the time series through integration: DFA without integration was more sensitive to the introduction of white noise and it showed significant predictive power to forecast the development of T2DM, while the pretreated time series did not. (4) Robustness of an interpolation algorithm for missing values: The modified DFA algorithm evaluates the percentage of missing values in a time series. Establishing a 2% error threshold, we estimated the number and length of missing segments that could be admitted to consider a time series as suitable for DFA analysis. For comparison with other metrics, a Principal Component Analysis was performed and the results neatly tease out four different components. The first vector carries information concerned with variability, the second represents mainly DFA alpha exponent, while the third and fourth vectors carry essentially information related to the two “pre-diabetic behaviours” (impaired fasting glucose and impaired glucose tolerance). The scaling exponent obtained with the modified DFA algorithm proposed has significant predictive power for the development of T2DM in a high-risk population compared with other variability metrics or with the standard DFA algorithm.

## Introduction

Many biological systems display a complex behaviour, and often one of the earliest signs of disease or senescence is the loss of complexity in its output [1–3]. This phenomenon may precede the first clinical signs and may have important prognostic implications.

The glucoregulatory system is a clear example. Glucose blood levels need to be tightly controlled while the patient operates in very different conditions. In the fasting status, hepatic glucose production is the main source of glucose available. On the other hand, in the postprandial state, the patient must handle an important glucose overload by using it as metabolic fuel or by storing it as glycogen. This balance is reached through a complex network of hormones with both feedback and feed–forward loops, and thus a potentially ideal field to explore complexity metrics.

During the last decades, T2DM (type 2 diabetes mellitus) has become a growing epidemic due to the rising prevalence of obesity as a consequence of the Western lifestyle, and is responsible for significant morbi–mortality among high–risk populations [4]. Nevertheless, considering T2DM as a disease derived only from high plasma glucose levels is an oversimplification: glucose oscillations may have adverse effects, independently of the raw glycaemic levels, and indeed glycaemic variability was shown to be a risk factor for glycaemic complications [5, 6].

The route from health to T2DM is a continuum that typically follows one of two possible paths. Some patients are able to maintain normal glycaemia during fasting but are unable to handle the glucose overloads induced by eating (impaired glucose tolerance), while other patients can handle these overloads, but are unable to lower their basal glycaemia to normal levels during fasting (impaired fasting glucose) [7]. Both mechanisms eventually merge together and coexist in full–blown T2DM. As the glucoregulatory dysfunction evolves, there is a progressive loss of the pancreatic beta–cell (responsible for the insulin secretion) and the risk of developing clinical complications gradually increases [8]. For clinical purposes, glycaemic thresholds have been established in order to classify patients by stages. Nevertheless, these thresholds are somehow arbitrary, and by the time a diagnosis of T2DM is made, 50% of beta–cell function has been lost [9, 10], patients have already developed end–organ damage and are at increased risk for cardiovascular disease [8, 11].

In the last years, the introduction of Continuous Glucose Monitoring Systems (CGMS) that allow for prolonged, high–frequency, innocuous assessment of interstitial glucose, has fuelled the development of new metrics to monitor glucose dynamics [12–16].

There is no general consensus on how to analyse glucose time series. Probably the most generalized metrics are just a distribution description (range, standard deviation, coefficient of variation). However, while simple and straightforward, these metrics have several drawbacks, the most important of them being the assumption of independence of points, and therefore omitting a crucial aspect of a time series: namely its sequentiality. Other metrics (Mean Amplitude of Glycaemic Excursion (MAGE) [17], Continuous Overall Net Glycaemic Action 2 hour (CONGA-2) [18], etc.) take into account the sequentiality but depend heavily on some arbitrary thresholds.

Complexity analysis seems an excellent tool to analyse glucose time series, and among the several possible approaches, DFA [19] is arguably the most frequently used. DFA alpha scaling exponent is higher (lower complexity) in type 1 diabetics than in healthy patients [20], and also increases as patients walk their path from health, through prediabetes to full–blown T2DM [2]. Furthermore, this metric may help predicting the risk of development of T2DM in at-risk populations [21].

The main idea underlying DFA is to analyse how the informational content of a time series is distributed throughout the different time–windows. A complex time series will have proportionally more information encoded in small time–windows. As the system decays, its ability to detect and react to minor changes is blunted, and thus the informational content of small time–windows decreases. This is manifested as an increase in DFA scaling exponent, that is, a decrease in its complexity.

In the present paper we propose a customization of the standard DFA algorithm for diabetes risk forecasting to achieve a significant classification capability. We review some of its characteristics and compare the performance of the simplified DFA algorithm with that of several other CGMS’ derived metrics [22], including other complexity statistics such as Approximate Entropy (ApEn) [23], Sample Entropy (SampEn) [24], or Poincaré plots [25]. To evaluate the performance of the complexity metrics we assessed their power to predict the evolution to T2DM in a population of patients with essential hypertension, a condition known to be a risk factor for T2DM development [26].

## Materials and methods

### Patients data set

The data set has already been published by our group [21]. Essentially, there were 208 patients selected from the outpatient clinic of hypertension and vascular risk of the University Hospital of Móstoles, in Madrid, from January 2012 to May 2015. The inclusion criteria were: age greater than 18 and lower than 85 years, a previous diagnosis of essential hypertension and the exclusion of a previous diagnosis of diabetes mellitus or treatment with antidiabetic drugs. The original study was approved by the Hospital’s Ethical Committee and a written informed consent was obtained from each patient before inclusion. A CGMS (iPro, MiniMed, Northridge, CA, USA) record was obtained at inclusion, for at least 24h with sampling every 5’. Patients were then followed every 6 months until the diagnosis of T2DM or end of study. A diagnosis of T2DM was established with either basal glucose tests ≥ 126mg/dl, and/or haemoglobin A1c test ≥ 6.5%, both confirmed in a second measurement. The median follow–up was 33 months (range: 6 to 72 months). There were 17 new cases of T2DM, with a median time to diagnosis of 33.8 months (IQR 24.1). Details may be found in Table 1.

**Table 1:** Baseline characteristics of included patients.

Gender (male/female)	103/105	
Variable	median	IQR
Age(Years)	61	12
Follow up (months)	33.1	19.2
BMI(Kg/m^2^)	29.3	5.4
Basal glycaemia (mg/dL)*	100.6	11.4
HbA1c(%)	5.8	0.4
Coefficient of variation	0.15	0.08
CONGA-2	19.11	11.8
MAGE(mg/dL)	39.5	21.9
Fluctuation index	12.25	7.4
TU100(%)	0.52	0.42
AO140	9	171.2
ApEn*	0.39	0.09
SampEn*	0.32	0.1
DFA *α* exponent*	0.9	0.09
Poincaré–SD1	1.52	0.67
Poincaré–SD2	23.4	12.8
Poincaré–E	15.5	5.2

IQR: interquartile range; BMI: body mass index; HbA1c: haemoglobin A1c; CONGA2: Continuous Overall Net Glycaemic Action 2 hour; MAGE: Mean Amplitude of Glycaemic Excursions; FI: Fluctuation Index; TU100: Time under the 100 mg/dl glycaemic threshold; AO140: Area over the 140 mg/dl glycaemic threshold; ApEn: Approximate Entropy; SampEn: Sample Entropy; DFA: Detrended Fluctuation Analysis *α* exponent; Poincaré–SD1: Standard deviation of points in the width axis of an ellipse fitted to a Poincare plot; Poincaré–SD2: Standard deviation of points in the length axis of an ellipse fitted to a Poincaré plot; Poincaré–E: Eccentricity of the ellipse (SD2/SD1).

* Variables with normal distribution are expressed as mean and standard deviation.

### DFA algorithm

The DFA script is based on the description in [19] and is written in R. It is publicly available at the journal’s site.

The input is a vector with at least 288 values (assuming a glucose measurement every 5’ during at least 24 hours), with no missing values at the beginning or the end of the series. Missing values are allowed along the series, and are handled as described later. If the time series has more than 288 values, the script goes through the whole time series, analysing every 24h segment with a 1h step (and 23h overlap) (e.g.: day 1: 08.00 to day 2: 08.00; day 1: 09.00 to day 2: 09.00, etc). Finally, the algorithm returns the sweeping average of DFA alpha exponent. The essential steps are:

- Missing values are repaired through linear interpolation [27]. In order to handle their weight, a *shadow variable* is created, stating which values are real measurements and which are interpolated. A tolerance threshold is established (by default, 0.2).
- The windowing is set to 3, 4, 6, 8, 9, 12, 16, 18, 24, 32, 36, 48, 72, 96, 144 and 288 points (all exact divisors of 288), and corresponding to time windows of 15’, 20’, 30’, 40’, 45’, 1h, 1h20’, 1.5h, 2h, 2h40’, 3h, 4h, 6h, 8h, 12h and 24h.
- For each time frame, the series is divided in segments, and a linear regression is calculated for each segment. The area between the linear regression and the real time series is evaluated:
  $$\begin{array}{c} F\left(n\right)=\sqrt{\frac{1}{N}\sum_{k=1}^{N}{\left[y\left(k\right)-y_{n}\left(k\right)\right]}^{2}} \end{array}$$
  where *F*(*n*) measures the difference between the time series and the regression line, *N* is the total number of data points, *y*(*k*) is the value of the pre–processed time series **y** at point *k* and *y*_*n*_(*k*) is the value of the regression line at that point. For each segment, the script evaluates if the number of interpolated values is greater than the tolerance threshold. If this is the case, that segment’s result is substituted by the mean of the segments of the same window length.
- This process is repeated for all time–window sizes, obtaining as many *F*(*n*) as time–window sizes.
- A linear regression model is fitted between log(*F*(*n*)) and log(window size). If the model fits correctly (*p* < 0.001), the alpha scaling exponent is obtained as the slope of the regression model; otherwise, a warning appears.

#### Windowing

To analyse the influence of the different windowing sets on DFA alpha’s predictive power, a vector with all the exact divisors of 288 was built. Every possible set of continuous time windows were tested, with a minimal window ranging from 15 minutes to 90 minutes and a maximal window ranging from 4 hours to 24 hours. With each of these windowing sets we calculated the alpha exponent value of the patients included in the data set, and evaluated its efficiency in predicting the development of T2DM. This was assessed by means of a Cox proportional hazard model (survival R package).

#### Coefficient of variation

In the cases when time series longer than 24h were available, a test was performed in order to explore the influence of the starting time of the 24 hour record. From 208 patients, 191 patients had a 48 hour CGMS record. In these patients, the alpha scaling exponent was calculated in sweeping 24h long segments, with 1h steps (and 23h overlap). A coefficient of variation was obtained for each patient.

#### Pre–treatment through integration

Most publications using DFA in diabetes treat the time series through integration before starting the detrending steps. This is usually performed through:

$$\begin{array}{c} y\left(k\right)=\sum_{i=1}^{k}\left(x_{i}-x_{\text{mean}}\right) \end{array}$$

where *x*_*i*_ is the value of the original time series **x** at point *i*. This ensures a brownian dynamic, allowing its inclusion in a *random walk* model, and standardizes the meaning of its results (e.g, *α* > 1.5: positive correlation, *α* < 1.5: negative correlation). Furthermore, this preprocessing smoothens the time series profile and solves the missing–values problem. However, this may significantly affect DFA’s sensitivity. In the present work, we propose to skip the pre–processing of the time series and proceed to the DFA calculation on the *raw* data, that is, **y** = **x**.

To provide a better support to our decision of not integrating the time series, we applied a method to ensure our CGM records could be considered as fractional Brownian motion (fBm) [28, 29].

To compare both methods (DFA with and without pre–treatment) we calculated both metrics in all our patients, and used both results in two different tests:

- *Influence of white noise*. Each of the first 24h of all the 208 time series included in the patient database were evaluated. From each time series, 15 replicas were created, with an increasing addition of random noise (uniform distribution with average 0 and range increasing from -1:+1 mg/dl to -15:+15 mg/dl). From each of the replicas, an integrated series was obtained following the above-mentioned algorithm. The two DFA metrics were calculated for each time series and were plotted against the intensity of the random component. A linear correlation model was fitted between the alpha exponent and range (random component), and the slope of the changes in both DFAs were compared.
- *Predictive power*. We compared the predictive power of the integrated time series vs. the non–integrated series in a Cox proportional hazard model [30] to forecast the development of full–blown T2DM.

Except for the sections dealing with the influence of pre–treatment through integration, all other alpha exponent results and figures referred in this manuscript were obtained omitting the aforementioned pre–treatment. For comparisons, henceforward *DFAint* and *DFAraw* are used to refer to DFA with or without integration, respectively.

#### Robustness of the interpolation algorithm

As exposed earlier, the DFA algorithm evaluates the percentage of missing (and thus interpolated) values. Although recent technological developments are minimizing missing data, this has been a limitation in the clinical context where conditions cannot be completely supervised.

DFA requires a time series without missing data. The trivial approach to missing values is interpolation. However, depending on the length and the number of missing segments, this may have serious effects on DFA results. Therefore, it seemed mandatory to assess the consequences of the length and number of missing (and interpolated) values, and to establish a threshold above which the time series can not be trustfully analysed. In order to do this, 30 time series with no missing values were selected. In each time series, 540 replicas were created and:

- A set of *n* segments of length *l* were deleted (and interpolated). The length of the omitted segments varied between 4 elements (20’) to 30 elements (150’) and the number of segments varied between 1 and 20 (or half the length of the time series, whatever happened before).
- The precise location of the missing segments was random, and the process was repeated 30 times for each time series.
- The mean absolute difference between the interpolated time series and the original series was evaluated for each combination of *n* (number of missing segments) and *l* (length of the missing segments).
- This process was repeated for all 30 patients, and a mean absolute error was calculated for each combination of *n* and *l* of the complete set of patients.

### Comparison of different metrics

We compared the efficiency (in predicting the risk of T2DM in a Cox proportional hazard model) of several of the most frequent CGMS variability metrics. An exhaustive discussion of each metric is out of the scope of this paper, but schematically:

1. ***Conventional*** **variability metrics**: coefficient of variation (standard deviation/mean).
2. **Variability metrics considering sequentiality**:
  - CONGA-2 [18] is the standard deviation (sd) of the range of differences in glycaemia between points separated by 120 min.
  - MAGE [17]. A *glycaemic excursion* was defined as a excursion greater than 1 SD of the time series.
  - Glycaemic Fluctuation Index [31]. The area between the glycaemic profile and the mean glycaemia.
3. **Metrics related with prediabetic phenotype**:
  - Time under the 100 mg/dl glycaemic threshold (TU100). Generally, the *Impaired Fasting Glucose* (IFG) phenotype is defined as a categorical variable (fasting glycaemia > 100mg/dl). However, transforming it into a quantitative variable may drastically change its sensitivity [32], so the percentage of time with glycaemia lower than 100 mg/dL has been measured as an indicator of fine fasting glucose control.
  - Area over the 140 mg/dl glycaemic threshold (AO140). Similarly, the *Impaired Glucose Tolerance* (IGT) phenotype is usually employed as a qualitative variable (glycaemia ≥ 140 mg/dL and < 200 mg/dL, 2 hours after 75-gr glucose overload). Again, transforming it into a quantitative variable (area above 140 mg/dl threshold) greatly increases its predictive value [32].
4. **Entropy-related estimations**. In essence, these metrics assess the *predictability* of the time series, evaluating to what extent the preceding points determine the following values. This is performed analysing the frequency of repeated patterns. Higher ApEn or SampEn, therefore less predictability, implies higher complexity:
  - ApEn [23].
  - SampEn [24].
5. **Metrics derived from the Poincaré plot** [33]. A plot is created with glycaemia at point (*i*) in the horizontal axis vs. glycaemia at point (*i* + 1) (*delay map*). The resulting cloud of points is fitted to an ellipse:
  - Standard deviation of the points on a delay map with respect to the horizontal axis (width) of the fitted ellipse (SD1).
  - Standard deviation of the points on a delay map with respect to the vertical axis (length) of the fitted ellipse (SD2).
  - Eccentricity of the fitted ellipse (E): SD2/SD1
6. **DFAraw**, as previously described.

ApEn and SampEn were calculated by means of the pracma R package. All other scripts were directly written in R. Among the various metrics with statistically significant predictive power, a Principal Component Analysis (PCA) (psych R package) was performed in order to assess how these metrics correlated with one another. In PCA, it is conventional to select only those factors with eigenvalues greater than 1, as an eigenvalue of 1 indicates that a factor can only explain as much variance as a single item [34]. For this reason, factors were selected if their eigenvalue was greater than 1.0. A varimax rotation was applied to clarify the structure of the loading matrix, and the rotated components (RCs) were used instead of the original principal components (PCs) since they provide a more intuitive interpretation of the results.

### Statistics

Variables are expressed as mean and standard deviation (SD) if they have a normal distribution, and as median and interquartile range (IQR) otherwise. All statistics were performed with R (https://www.r-project.org). Significance was assumed when 2–tail *p* < 0.05 (except for the log(Fn)–log(window) correlation in DFA algorithm, in which, this being a log–log correlation, a *p* < 0.001 was required).

## Results and discussion

In the present work we introduce several variations of the DFA algorithm that could provide some advantages regarding its applicability in the clinical field. Moreover, we test its accuracy on a practical scenario by correlating its results with proportional risk of T2DM development in a high risk population in real–life conditions, followed up for a period of time.

### DFA windowing

**Fig 1:**
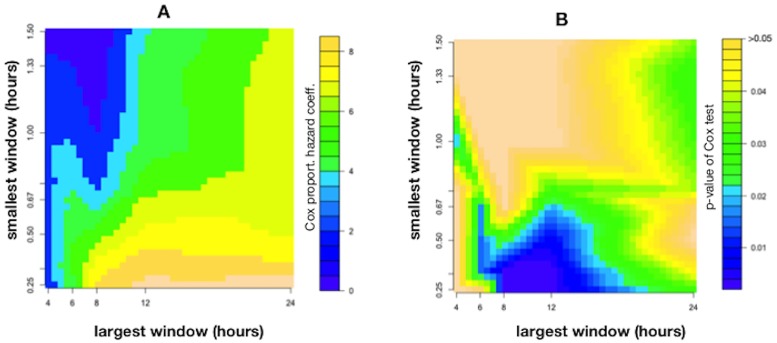
Predictive power of DFA alpha scaling exponent to forecast the development of T2DM on Cox survival analysis. (A) Heatmap with Cox proportional hazard coefficient for different windowings. (B) Cox coefficient’s p-value for different windowings.

Among all the windowing sets evaluated, the best predictive value for T2DM development was obtained with the set that included all time-windows from 15’ to 24h (15’, 20’, 30’, 40’, 45’, 1h, 1h20’, 1.5h, 2h, 2h40’, 3h, 4h, 6h, 8h, 12h and 24h), with a Cox coefficient of 8.344(*p* = 0.027). A 0.1 increase in DFAraw alpha scaling exponent results in 2.3 odds ratio of T2DM. Other sets of windowing (e.g. from 20 minutes to 24 hours; from 15 minutes to 12 hours) provided smaller Cox coefficients and therefore they do not seem to offer any advantage. Fig 1 displays two heatmaps with the Cox coefficient for different windowings and the p-values of the Cox model.

### Coefficient of variation

As explained before, DFA alpha exponent was calculated on sliding 24-hour segments with 1-hour steps for those series longer than 24 hours, and a coefficient of variation was obtained for the values of each patient. The median coefficient of variation was 3.2%, with an interquartile range of 2.2%. Our data show no significant differences in alpha values between different segments of 24 hours time series taken at different day-times. It has been well-established that there is a level of regulation based on circadian rhythms beyond sleeping patterns and meals. Many researchers have underlined that the plasmatic glucose rhythm may be influenced by hormones whose secretion is narrowly related to day–night rhythm (such as cortisol, growth-hormone, etc. [35, 36]). However, our results suggest that analysing time series of at least 24h avoids the influence of circadian rhythms and thus the results are not significantly interfered by the day–time in which the CGMS is implanted.

### Pretreatment through integration

Although most authors using DFA in diabetes integrate time series before detrending, this is not universally accepted, and some authors have applied DFA without pre-integration [37]. Despite the extended practice, we feel that this pre–processing may have a great cost in sensitivity.

The power spectra of all the time series was computed, and a non-linear *y* = *α* * *x*^*β*^ power curve was fitted (*p* < 0.01 in all cases) to the frequency domain data in order to estimate the exponent *β*. Averaging all the results obtained, the exponent value achieved was *β* = −8.144719 + / − 0.5175, which is clearly in accordance with the fBm assumption, and therefore we assumed that integration could be omitted [28].

**Fig 2:**
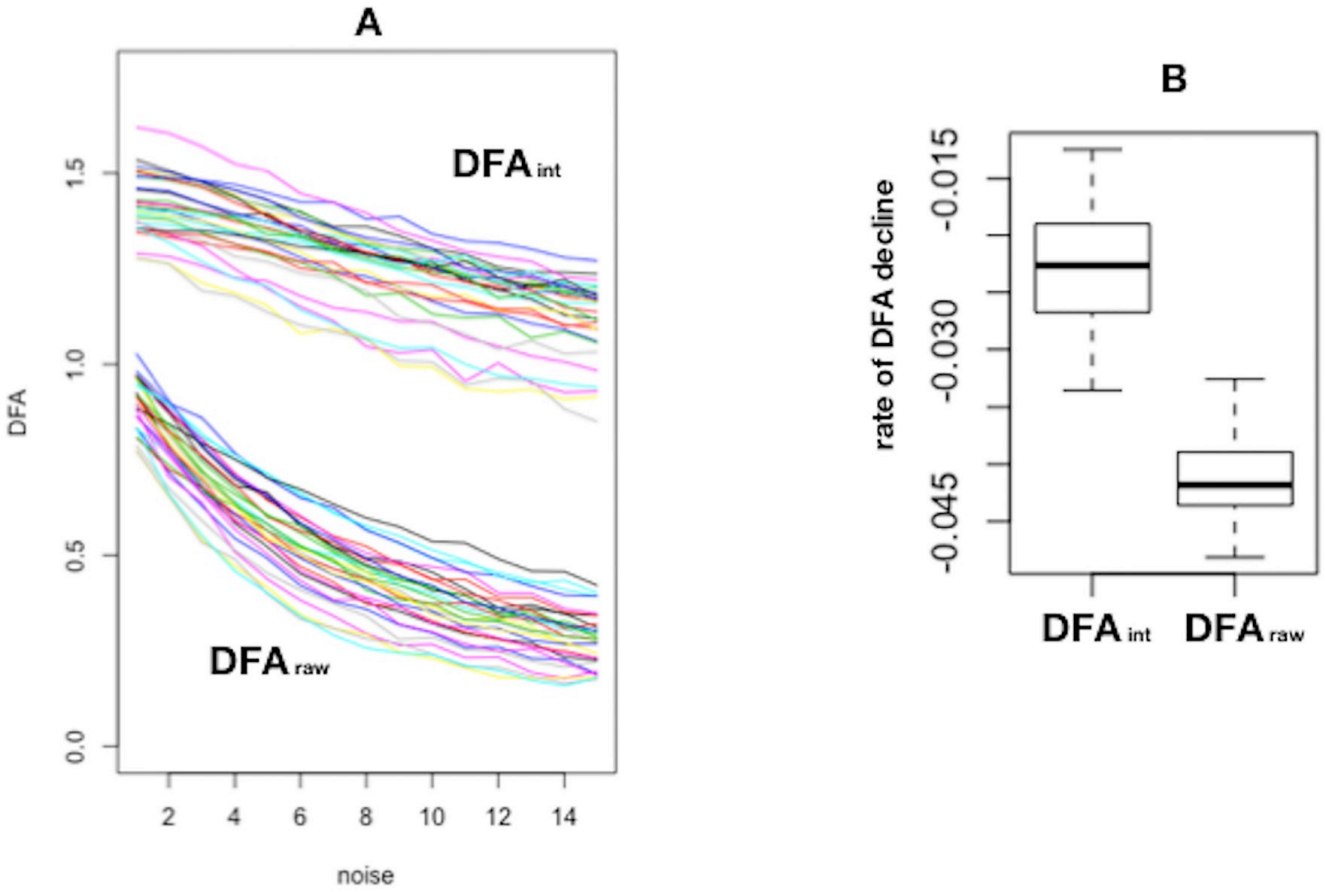
Influence of integration on DFA alpha scaling exponent values. (A) Rate of decline of alpha with the addition of an increasing component of randomness (*white noise*) in series pre–treated through integration (DFAint) or not (DFAraw). (B) Boxplot of the differences of the alpha exponent values between both methods.

To evaluate the influence of pretreatment through integration before proceeding to detrending, two tests were performed:

- **Influence of a random component (white noise)**. Considering that white noise has *α* = 0.5, we assumed (and empirically confirmed) that introducing white noise in the time series would reduce its alpha exponent. In order to determine to what extend alpha value is altered by white noise, each time series was exposed to a random component as explained in the Material and Methods section, to create several replicas of the series and alpha exponent was measured in each replica, both with and without pre–treatment through integration. The rate of decline was significantly steeper in non-integrated than in integrated series (-0.042 (SD: 0.0033) vs. -0.023 (SD 0.0053); *p* < 0.001). Fig 2 displays the evolution of DFAraw and DFAint as the intensity of the random component increased.
  More formally, a General Linear Model was built with DFA alpha exponent as the dependent variable and the intensity of the random component and pre–treatment through integration (qualitative: yes/no) as independent variables. As expected, both variables were statistically significant (integration: *coef*. = 0.627, *p* < 0.01; randomness: *coef*. = −0.041, *p* < 0.019) but notably so was their interaction (*coef*. = 0.016, *p* < 0.01). This data show an earlier and steeper drop of alpha as the random component increases in the non-integrated time series compared to the pre-treated time series. This suggests a decrease in sensitivity attributable to the pre–treatment through integration.
- **Predictive power**. As stated previously, in the non–integrated time–series, several windowing sets produced significant predictions, with a maximum Cox coefficient of 8.344 (*p* = 0.027). In contradistinction, no set of windowings was able to produce a significant Cox model with the integrated time–series.

To summarise, our data suggest that omitting integration and thus stepping out of the *random walk model* increases alpha’s sensitivity and may arguably boost its clinical efficiency. Of course, the standard 1.5 threshold loses its meaning, but DFA remains a useful tool to explore how the informational content of a time series is distributed throughout its time–windows, and may be employed to compare different glycaemic profiles.

### Robustness of the interpolation algorithm

To explore the influence of missing (and interpolated) values on the scaling exponent calculations (i.e. to evaluate the robustness of the DFA algorithm in handling lost measurements), we deleted an increasing number of randomly distributed segments of increasing length and assessed the resulting error (compared with the complete series), as exposed above.

**Fig 3:**
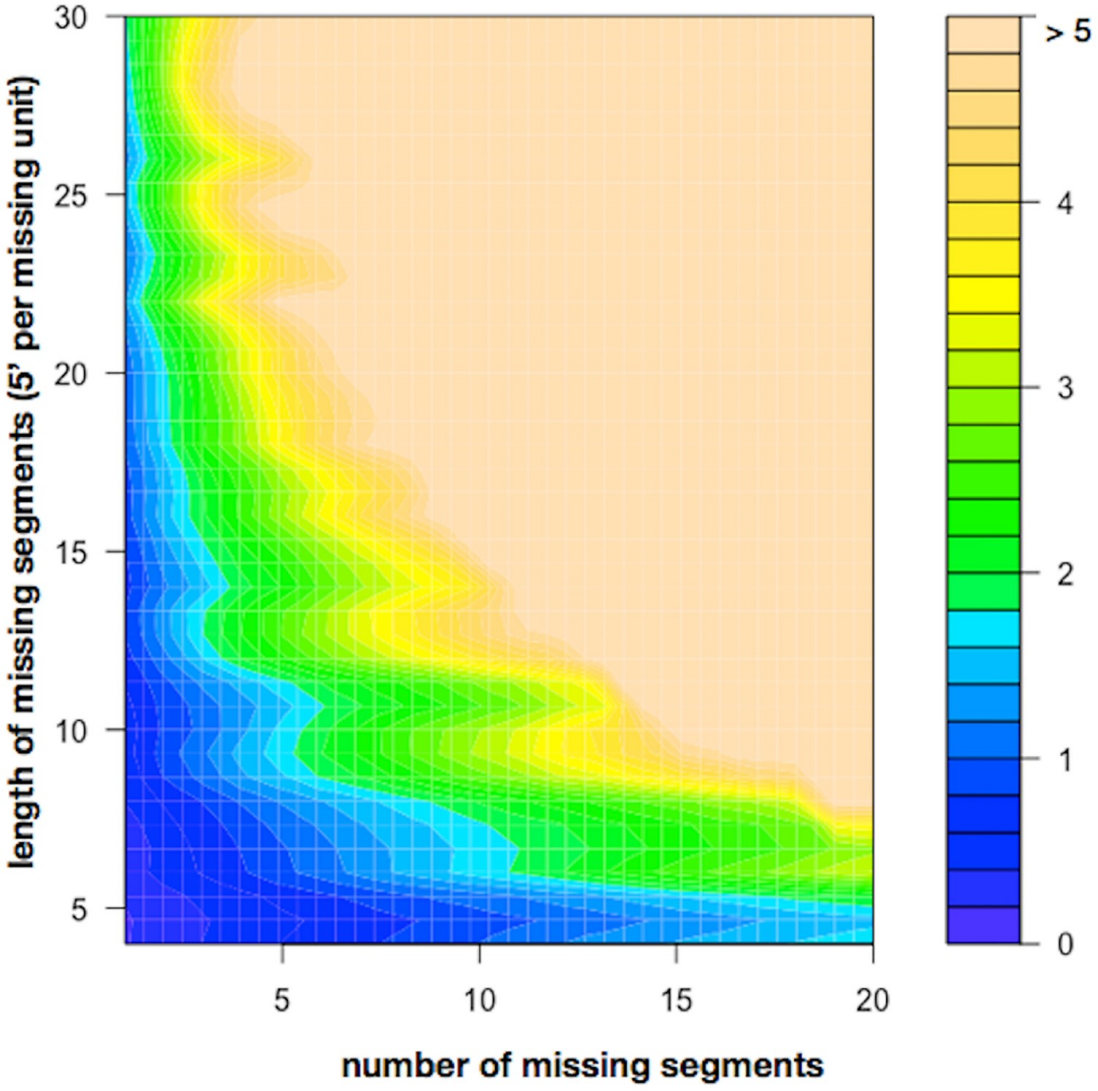
Error depending on the number and length of missing segments. Each unit of missing segment represents 5 minutes. In 30 series originally with no missing values, an increasing number of randomly distributed segments of increasing length were deleted and interpolated. The process was repeated 30 times for each combination of length and number of deleted segments and for each patient, and DFA scaling exponent was calculated for each replica. The mean error (absolute difference with *the real alpha value* (complete series)) was recorded for each combination of length and number of missing segments.

Fig 3 is a heat–map representing the error (as %) depending on the number and length of missing segments. We fixed a threshold of 2% error to consider a time series as suitable for DFA analysis. With this limit, we propose an *admission criteria* for a time series to be subjected to DFA: schematically, a time series should be rejected if ever the missing segments surpassed: twenty times a 20’ segment, or eleven 30’, or six 45’, or four 1h, or one 1.5h, or any longer than 1.5h missing segments. To ensure compliance, we suggest counting the number of missing segments and establishing the length of the largest missing segment.

### Comparison of different metrics

Several CGMS–related metrics (described above) were applied to the first 24h series of all patients, and their results were included as independent variables in a univariate Cox proportional hazard model to assess the risk of T2DM development. Results of this analyis are displayed in Table 2. Only CONGA2, MAGE, TU100, AO140, Poincaré–SD1, Poincaré–SD2 and DFAraw alpha exponent were significant predictors.

**Table 2:** Cox proportional hazard model for different metrics.

Metric	Cox coefficient	*p*
Coefficient of variation	2.679	0.499
CONGA-2	0.061	**0.004**
MAGE(mg/dL)	0.019	**0.003**
Fluctuation index	0.082	0.065
TU100(%)	-3.05	**0.004**
AO140	0.0006	**<** **0.001**
ApEn	-2.671	0.341
SampEn	-0.767	0.741
DFAraw *α* exponent	8.344	**0.027**
Poincaré–SD1	0.781	**0.013**
Poincaré–SD2	0.05	**0.03**
Poincaré–E	0.006	0.92

To analyse how these variables related with one another, we performed a PCA with varimax rotation as referred in Material and Methods. Table 3 presents the resulting model. Four principal components were selected, carrying 96% of the variance. The results neatly tease out four different elements. The first vector (RC1) carries information concerned with variability. The vector carrying the second largest amount of variance (RC3) represents mainly DFAraw scaling exponent, while the third and fourth vector carry essentially the information related with the *two pre–diabetic behaviours* (impaired fasting glucose in the third vector, RC4, and impaired glucose tolerance in the fourth vector, RC2).

**Table 3:** Principal Components Analysis of the variables selected in the Cox proportional hazard model.

A	RC1	RC3	RC4	RC2
SS loadings	3.09	1.47	1.09	1.05
Proportion of variance	0.44	0.21	0.16	0.15
Cumulative variance	0.44	0.65	0.81	0.96
B	RC1	RC3	RC4	RC2
CONGA-2	**0.84**	0.41	0.26	-0.14
MAGE(mg/dL)	**0.80**	0.42	0.25	-0.08
TU100(%)	-0.14	-0.11	-0.17	**0.97**
AO140	0.34	0.15	**0.90**	-0.21
DFAraw *α* exponent	0.22	**0.95**	0.12	-0.11
Poincaré–SD1	**0.95**	-0.09	0.15	-0.15
Poincaré–SD2	**0.81**	0.42	0.29	-0.07

Loadings greater than 0.8 are highlighted. (A) SS loadings for each of the components. (B) Standardized loadings for each variable. RC 1–4 refers to each of the rotated (Varimax) components obtained by PCA.

The prompt separation of variability metrics (first vector) and DFA scaling exponent (second vector) probably reveals that, although in several studies [2, 21, 32] variability and complexity show a strong inverse correlation, they explore different phenomena. Arguably, variability metrics deal with short–term fluctuations, and are less sensitive to long–term correlations. Instead, DFA explores this issue analysing the correlation behaviour as the time window is modified, and thus exploring the informational content all along different *time–grainings*. It seems reasonable to expect that physiologic systems based on complex hormonal loops (sometimes requiring sensing, protein synthesis, transportation and distant action) should need a wide variety of time–windows to be fully explored.

## Conclusions

We propose and analyse a DFA algorithm that omits pre–treatment through integration and manages missing (and interpolated) data considering their weight in each time window. This algorithm has significant predictive power as for the development of T2DM in a high–risk population. Furthermore, this metric explores dynamical aspects of the time series not displayed by other variability metrics.

Omitting pretreatment through integration improved substantially the predictive power of DFA alpha exponent (best Cox coefficient with optimal windowing: 8.32, *p* = 0.03), while no set of time-windows had significant predictive power with pre-treated time series. We describe the effect of different number and size of missing segments, and we propose an *admission criteria* to assure an error les than 2%. Several glycaemia-related metrics are able to predict the development of T2DM in our population. A principal component analysis on these metrics neatly teases out four vectors: one grouping variability metrics (CONGA–2, MAGE and Poincaré plot–derived variables), another displaying complexity (DFA alpha exponent) and two other displaying the two polar prediabetic phenotypes (impaired glucose tolerance and impaired fasting glucose).

DFA alpha scaling exponent (omitting pre–treatment through integration) has significant predictive power on the development of T2DM in patients at risk, independently of other variability metrics.

The main limitation of our study is the inability of comparison with most DFA studies due to our decision to omit pre–treatment through integration. Furthermore, the differences in the measurements between different glucometers are unknown. For that reason, we believe more research should be done in order to test this algorithm in different populations, with bigger samples and different glucometer models.

## Supporting information

S1 File Glucose time series and clinical database. This file includes glucose time series from all patients included in the study and the database with clinical variables.
(ZIP)

S2 File DFA algorithm script. This file includes the modified DFA algorithm script written in R.
(R) Click here for additional data file.
